# Life Cycle Assessment Comparison between an Earthbag Building and a Conventional Sahrawi Cement Blocks Building

**DOI:** 10.3390/ma17051011

**Published:** 2024-02-22

**Authors:** Ariadna Carrobé, Albert Castell, Ingrid Martorell

**Affiliations:** Sustainable Energy, Machinery and Buildings (SEMB) Research Group, INSPIRES Research Centre, Universitat de Lleida, Père de Cabrera s/n, 25001 Lleida, Spain; ariadna.carrobe@udl.cat (A.C.); albert.castell@udl.cat (A.C.)

**Keywords:** earthbag, Cement Blocks Building, Life Cycle Assessment, SimaPro, Ecoinvent, ReCiPe2016, Western Sahara, sustainability assessment

## Abstract

Growing environmental awareness has prompted a resurgence in traditional building techniques that rely on natural or recycled materials since many believe that structures made from these resources are ecologically friendly. Using Life Cycle Assessment (LCA) for construction materials offers valuable insights into the impacts produced during their production and construction processes. This study aims to assess the environmental impacts of two different constructions—an Earthbag Building (EB) and a conventional Sahrawi Cement Blocks Building (CBB). It also determines whether it is more environmentally beneficial to construct traditionally, utilizing local materials and aligning with the principles of the circular economy, which is one of the Sustainable Development Goals (SDGs) in Europe. This study specifically examines a cradle-to-gate LCA, using the software Simapro v. 9.4.0.1. Results show that in 21 out of the 22 impact categories analyzed, the CBB performs worse, in some cases presenting an impact of 70% higher than the EB. The highest impact is obtained for fine particulate matter formation and Global Warming related to Human Health categories, whilst impact categories related to water consumption and eutrophication obtained an impact of less than 0.001 for both constructions.

## 1. Introduction

### 1.1. Life Cycle Assessment 

The construction industry exerts a notable influence on the environment, primarily due to its consumption of natural resources, energy utilization, and emissions of greenhouse gases (GHGs) [1]. One of the most important aspects, regarding the sustainability of a building, is the selection of construction materials [2]. The Life Cycle Assessment (LCA) is a relevant tool that facilitates society to understand the environmental impact of construction materials through their entire life cycle. LCA considers the environmental impacts produced by any material in the different parts of its life cycle, including extraction of raw materials, production, transportation, construction, usage, and disposal. The impact categories studied in the LCA go from GHG emissions to depletion of ecosystems, including, air pollution, waste generation, or water pollution, among others [3].

The Life Cycle Assessment (LCA) provides a comprehensive way to thoroughly assess the environmental consequences associated with construction materials [4]. By examining the complete life cycle of these materials, from their initial extraction to their eventual disposal, LCA offers an in-depth understanding of their environmental impacts, enabling decision-makers to pinpoint the most impactful areas and implement focused strategies for enhancements [5].

Another advantage of the LCA is that it simplifies the process of comparing and selecting construction materials based on their environmental performance [6]. By quantifying and evaluating different environmental impacts, such as resource depletion, energy usage, emissions, or waste creation, LCA empowers individuals to opt for more sustainable materials and promotes the integration of new sustainable practices within the construction sector.

Furthermore, LCA studies contribute significantly to the formulation of environmental policies and regulations within the construction sector. The scientific foundation, directed by the insights from LCA, aids governments and regulatory bodies in establishing benchmarks, guidelines, and certifications [7]. 

Finally, ensuring clear and concise communication of LCA outcomes to stakeholders is essential. LCA will provide awareness and comprehension to clients, investors, suppliers, and the general public [8], who will take action to transform the construction sector into a more sustainable environment for future generations.

### 1.2. LCA in Earthen Construction

The Life Cycle Assessment is highly supported for its ability to contribute to sustainable building development, particularly in the case of earthen construction [4]. Previous studies emphasized the necessity of employing environmental LCA to evaluate strategies for using earthen materials in construction [9]. 

Despite the widespread discussion regarding the environmental advantages of earthen construction, there is a limited number of thorough investigations into its actual environmental effects. 

Currently, the available studies on the Life Cycle Assessment (LCA) of earthen construction focus on assessing the environmental impacts of specific components such as adobe bricks [10,11], rammed earth structures [12,13,14,15,16,17,18], earth plasters [19,20], COB walls [21,22], CEB [23], or Earthbag Buildings [24]. 

However, the currently available research has several shortcomings:

- The lack of comparison with standard materials and techniques makes it difficult to draw eco-friendly guidelines.
- These individual Life Cycle Assessment (LCA) studies cannot be easily compared, because they use site-specific and material/process-specific data unique to each study.
- The lack of uniformity when considering the functional unit does not enable direct comparisons between different construction techniques. Functional units, such as 1 kg of material [10], 1 m^2^ wall [20,21], or specific samples like a brick [13,15], are found in the literature. In order to compare those techniques, a common functional unit should be used.

To address the gaps in knowledge about the environmental consequences of earthen construction, future research must be focused on assessing the environmental impacts of earthen construction, considering its materials but also the construction process of the whole building structure.

As concluded by Rincón, et al. [25,26] for hot climates, earthen constructions have great performance in terms of hygrothermal comfort. Moreover, among the earthen constructions, those constructed completely with earth, such as Earthbag, have better performance than adobe traditional dwellings with metal sheet roofs [26]. 

The aim of this paper is to compare two construction techniques currently used in developing countries: an Earthbag Building (EB) versus a conventional Saharawi Cement Blocks Building (CBB). Earthbag construction involves the use of earth as the primary material, with a dome-shaped self-supporting structure, minimizing the materials and elements required for its construction. This technique represents traditional construction, utilizing local materials and promoting the circular economy, one of Europe’s Sustainable Development Goals (SDGs). On the other hand, CBB construction with metal sheet roofs involves more industrial processes to obtain the basic construction materials, such as the cement blocks or the sandwich panel roof, as well as the transportation of those materials, resulting in a more industrialized building. 

This comparison aims to assess the environmental impacts of both construction methods and underscores the importance of more sustainable construction practices by using locally sourced materials and embracing a circular economy approach in the region. 

## 2. Materials and Methods

This work stems from the ongoing challenges faced in refugee camps of the Western Sahara, where its inhabitants are changing their construction methods, going from the vernacular architecture based on earth as the main material, to the use of metal and cement blocks for construction. In this region, the earth, as a construction material, can be found in the proximities of the site, whilst the cement and the other processed materials must be imported from other countries.

The climate in the Western Sahara, according to the Köppen climate classification, is categorized as a hot desert climate (BWh). This climate is distinguished for having episodes of extreme heat and severe drought, mainly during the summertime. However, in the refugee camp area, there are episodes of extremely intense rains that have consistently destroyed the dwellings of its residents over the years. 

That is the main reason why traditional earthen techniques were used for buildings, but nowadays for new constructions, they are mainly using cement blocks and other industrialized materials that must be imported from other countries. However, this type of construction does not exhibit the same hygrothermal behavior as earth-based constructions, and, in fact, a lower level of comfort is detected inside the buildings [24]. Furthermore, due to the necessary imports for its construction, it does not promote the use of local materials and, eventually, a circular economy model.

### 2.1. Buildings Description

In the following study, a comparison in terms of the LCA of two buildings constructed with different techniques is presented. In both cases, the dimensions of the buildings are equivalent so that it is possible to compare the environmental impacts of the whole construction.

On the one hand, there is an Earthbag Building, constructed with earth, and in a dome shape. It has a circular base with a 3 m diameter and a maximum height of 3.3 m, obtaining a net floor area of 7.07 m^2^ and a total inner volume of 17.67 m^3^. The Earthbag walls are 35 cm thick, while the buttress uses a double layer of earthbag having 70 cm of thickness. The Earthbag dome roof is approximately 28 cm thick on average. The bags, made of polypropylene (PP), are filled with an earth mixture consisting of 92.21% sand, 3.42% lime, 3.57% clay in weight, and a small portion of fine gravel (0.8%). The earth mixture is compacted manually. The exterior of the building is coated with a 4 cm thick layer of lime mortar. The floor is separated by a waterproof polyethylene (PE) plastic layer. The main door entryway is located facing south and measures 0.91 × 2 m. There are two windows facing east and west and measuring 0.8 × 0.67 m and 0.6 × 0.35 m, which represents a glazed area of 0.25 m^2^ and 0.21 m^2^, respectively. Both windows and the door have a wooden frame of 6 cm, resulting in 0.0266 m^3^ of wood. Since the Earthbag techniques require a dome shape for their construction, above the windows, there is a free space covered with polystyrene insulation (6 cm) and a 2.2 cm thick wooden layer (see the Earthbag image in Table 1). This building is representative of a traditional vernacular construction.

On the other hand, there is the Cement Blocks Building (conventional building) constructed with cement blocks of 40 × 20 × 15 cm. Since a conventional building does not have a dome shape but a rectangular one, the total inner volume has been considered as close as possible to the Earthbag Building (17.63 m^3^) for comparison purposes. The floor net area is 7.05 m^2^, with a dimension of 2.35 × 3 m and a total height of 2.5 m. In order to guarantee the stability of the building, reinforced steel is used in the total floor area and along the walls at 70 kg/m^3^ [27]. Between the cement blocks, 10 mm of cement mortar is needed to stick them together in both directions [28]. For the roof, an insulated sandwich panel made of two layers of 1 mm aluminum sheet and 40 cm thick polystyrene insulation is considered. The door and windows considered in the conventional building are the same as the Earthbag Building, so the analysis is not interfered with by their impact. In this case, there is no need to add the insulated polystyrene above the windows (see the Cement Blocks Building image in Table 1).

In Table 1, the principal characteristics of the constructions considered in this study are presented.

### 2.2. Scope

The scope of this paper is to conduct a Life Cycle Analysis using a cradle-to-gate approach for the two constructions described in the previous Section 2.1. The research is focused on comprehensively assessing and comparing the environmental impacts associated with these constructions, from resource extraction to the final construction phase. 

The analysis does not take into account the environmental impacts of manual construction tools like hammers, compacting rammers, buckets, hoses, or formworks, as these tools are assumed to be used in other constructions, and their impact on this construction is neglected. 

The materials considered for the Life Cycle Inventory (LCI) are those used for the construction of the buildings, according to their dimensions and construction processes. However, some construction processes need additional sources of energy, for which their exact amendments are unknown, for example, the exact hours of water pumping, the amount of diesel consumed by the excavator while doing the foundation, or the time the mixer is used to produce the mortar in both buildings. These missing details are estimated based on similar projects.

Additionally, the energy used for the overall assembly of the windows, doors, polypropylene bags, and sandwich panels is not considered in this analysis. Nevertheless, the energy and transportation impact necessary to produce the subassembly parts of those elements is considered. It is important to highlight that transportation is considered based on generic values provided by the software.

### 2.3. Life Cycle Inventory, Impact Assessments, and Simapro Software

The Ecoinvent 3 database has been used for the Life Cycle Inventory because it is renowned as one of the largest, most thorough, and transparent databases accessible. It covers more than 18,000 distinct sets of data, providing extensive information on diverse products, services, and processes. This database is adaptable to nearly all Life Cycle Assessment (LCA) approaches and is incorporated into well-known software tools like Helix, Mobius, Simapro, GaBi, and open LCA. This widespread compatibility makes it easily accessible and practical for a wide array of LCA professionals. 

In order to conduct the Life Cycle Assessment, each construction is divided into various assemblies. These assemblies can vary in complexity and may include one or multiple raw materials and processes. In some cases, these assemblies become more complex, leading to the creation of sub-assemblies. The unit “p,” as defined by the analysis software, indicates that in a given assembly, there is one unit of the corresponding sub-assembly included.

In Table 2 and Table 3 the Life Cycle Inventory for both constructions considered is presented. 

The Life Cycle Impact Assessment (LCIA) has been carried out using the ReCiPe 2016 Endpoint methodology [29]. This approach is commonly used and merges the strengths of the CML2002 and Ecoindicator-99 methods. Moreover, this methodology covers midpoint and endpoint impact categories, which makes it clear to identify and relate the different impact categories to the area that they affect the most. For example, the activity of removing earth as a raw material can produce a low impact on the use of natural resources; however, the way this excavation is carried out (burning diesel for the heavy machines) can have a significant impact on human health, due to the small particles released.

The software Simapro version 9.4.0.1 (created by PRé Sustainability) has been used to conduct both LCA and the comparison between the two different buildings. SimaPro stands as a widely utilized LCA software worldwide [30]. The software’s collection includes databases such as Ecoinvent, USLCI, ELCD, and Agri-footprint, among others. SimaPro follows the ISO 14000 standards [31] for conducting LCA studies and crafting diagrams and figures.

The results presented in the following section correspond to the midpoint and endpoint impact categories considered in the ReCiPe Endpoint methodology. Table 4 presents impact points for all impact categories, both for normalization and ponderation. However, for clearer visualization, in the following figures of Section 3, only the impact categories with an overall score higher than 0.05 pt are shown. The unit pt corresponds to the “Eco-Indicator point” being 1 pt one-hundredth of the annual environmental burden of an average European citizen [32].

## 3. Results and Discussion

**Figure 1:**
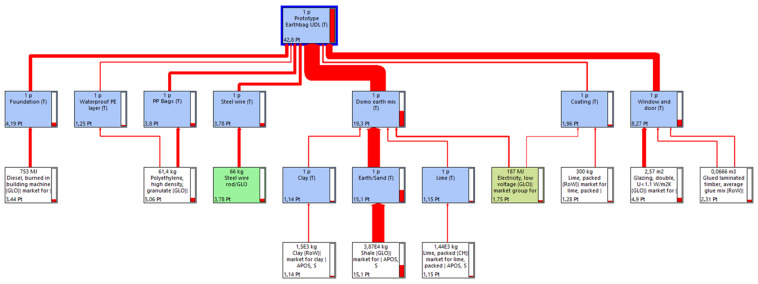
Flux diagram of the subassemblies of the Earthbag Building.

**Figure 2:**
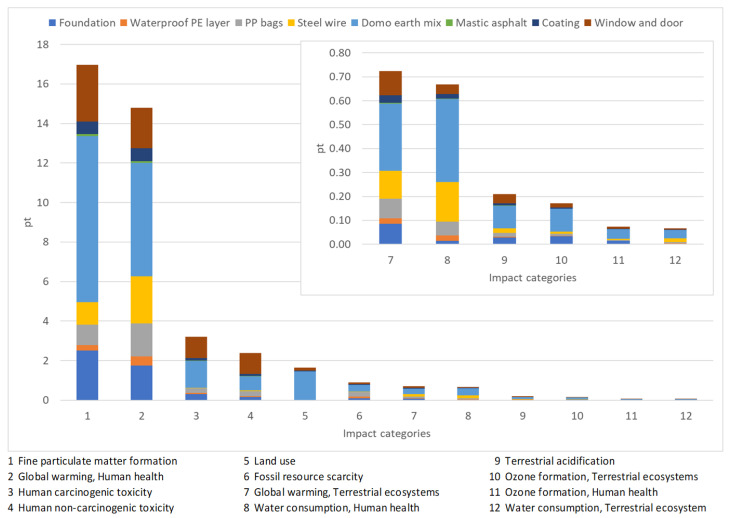
Ponderation of the subassemblies included in the Earthbag Building according to the different impact categories, considering higher impacts of 0.05.

**Figure 3:**
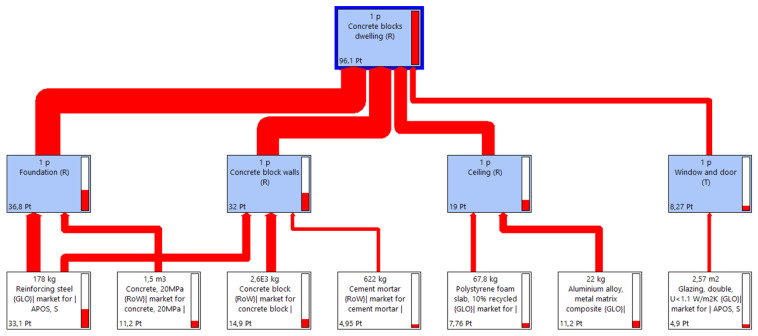
Flux diagram of the subassemblies of the Cement Blocks Building construction.

**Figure 4:**
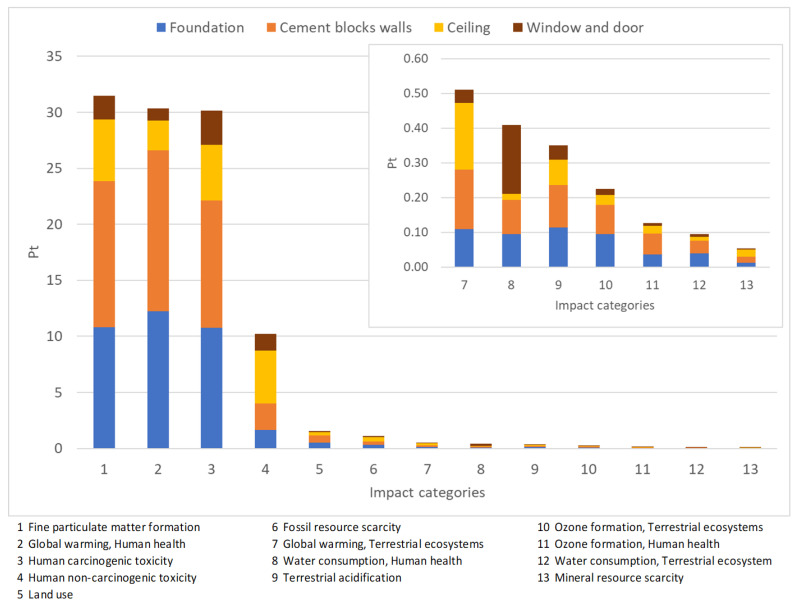
Ponderation of the subassemblies included in the Cement Blocks Building according to the different impact categories, considering higher impacts of 0.05.

**Figure 5:**
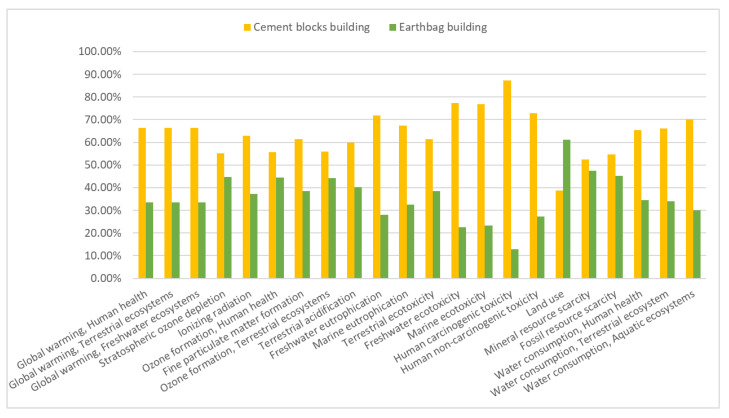
Characterization of both constructions according to the different impact categories.

**Figure 6:**
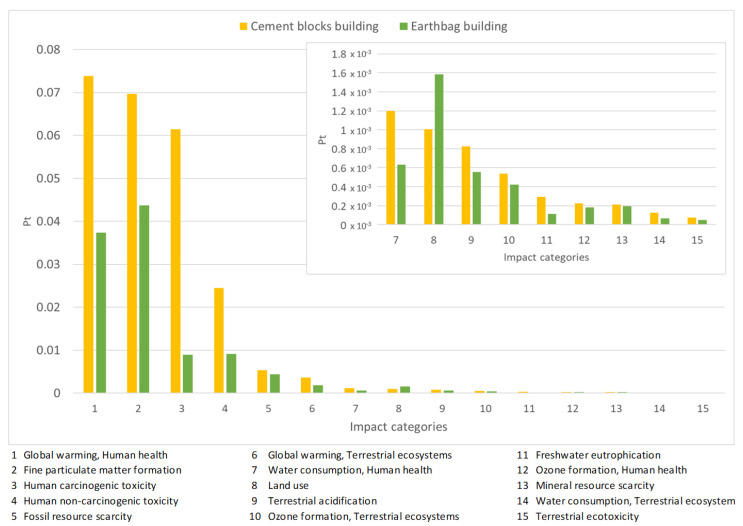
Normalization of both constructions according to the different impact categories, considering higher impacts of 0.05.

**Figure 7:**
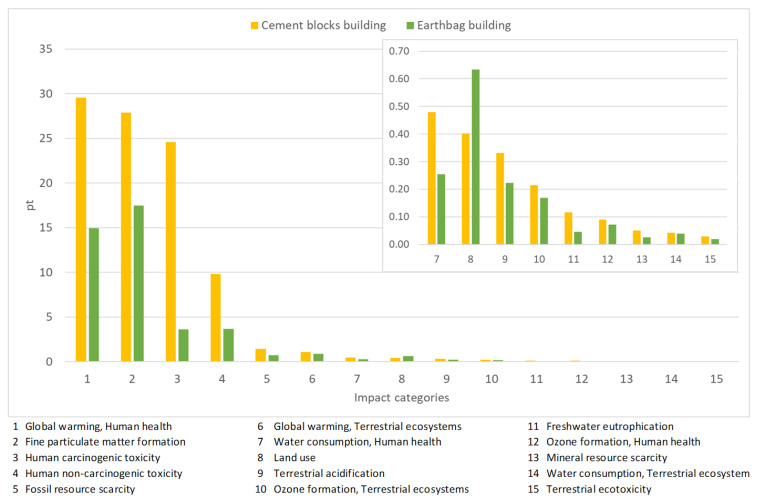
Ponderation of both constructions according to the different impact categories, considering higher impacts of 0.05.

**Figure 8:**
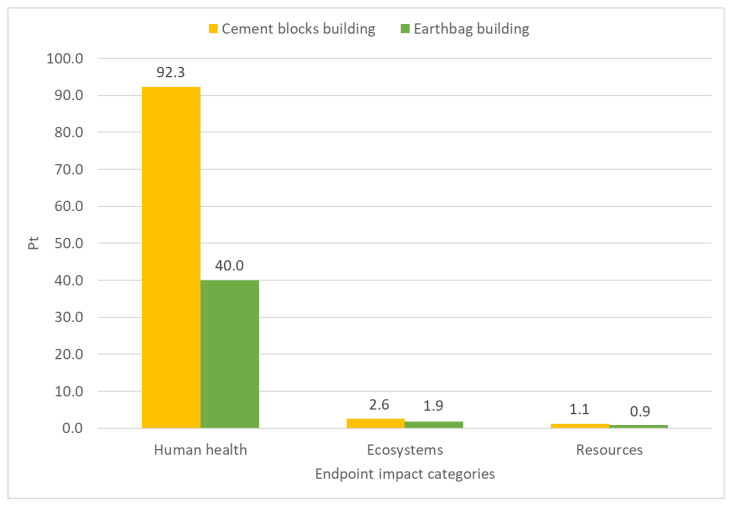
Ponderation of both constructions according to the different endpoint categories.

In order to compare the environmental impact of both constructions, an individual LCA has been conducted for each building: the Earthbag Building (EB) and the conventional Cement Blocks Building (CBB). Figure 1 and Figure 2 correspond to the EB construction, whilst Figure 3 and Figure 4 correspond to the CBB construction. From Figure 5, Figure 6, Figure 7 and Figure 8 comparative analysis of both constructions are shown.

In Figure 1 and Figure 3, the flux diagram of the Life Cycle Inventory of both constructions is presented. Those diagrams illustrate how each subassembly contributes to the overall building, along with the main materials and energy related to these subassemblies.

In Figure 1, the flux diagram of the Earthbag Building is presented. The materials used for its construction have been divided into eight subassemblies: foundation, waterproof PE layer, PP bags, steel wire, domo earth mix, mastic asphalt, coating, and doors/windows.

As expected, the greatest environmental impact contribution to the Earthbag Building corresponds to the earth mix subassembly used to fill the PP bags (45.1%). The door and windows subassembly follows the earth mix (19.3%), but in this case, its contribution to the whole building impact is not related to the volume of the subassembly but to the processed elements included in it, for example, the wooden frame and the double glassed surface or the polystyrene insulation above the windows. On the other side, due to the volume of material used, the less impacting subassembly corresponds to the waterproof PE layer (2.9%).

Figure 2 presents the impact of the EB subassemblies according to each midpoint category.

In Figure 2, it is observed that the subassembly with the highest impact in most of the midpoint impact categories is the domo earth mix since it represents almost half of the construction in terms of total weight, as presented in Figure 1.

Among all the midpoint impact categories, the domo earth mix subassembly presents the highest impact on the fine particulate matter formation impact category (8.5 points) because it is directly related to the particles released by the heavy machines during its extraction as a natural resource.

Nevertheless, when evaluating the impacts of global warming on human health, it becomes evident that certain processed materials have a higher impact] in proportion to their weight contribution. For example, despite the weight contribution to the EB construction of the steel wire subassembly, being 5.11 times smaller than the domo earth mix, its impact does not align proportionally; it is only 2.4 times smaller, which states that the processed materials have a higher impact on the overall construction. Similar results are obtained for other processed subassemblies like the windows and door.

In Figure 3, the flux diagram of the subassemblies involved in the Cement Blocks Building is presented. In this case, there are four subassemblies: foundation, cement blocks walls, ceiling, and windows and door.

As described in Section 2.1, most of the materials used in this construction are highly processed materials. The one having the highest environmental impact is the reinforced steel, which is used both in foundation and the cement blocks walls. Thus, those subassemblies have the highest environmental impact on the whole CBB, at percentages of 38.3% and 33.3%, respectively. The windows and door subassembly contribute the least to the conventional building, at a percentage of 8.6%.

In order to analyze which subassembly, included in the CBB, contributes the most to the different impact categories, the pondered results are presented in Figure 4.

As shown in Figure 4, the production of the cement blocks as well as the foundation are the two subassemblies with the highest impacts in most of the midpoint categories. Those subassemblies include high energy demands for their production and manufacture. Moreover, for the foundation, the diesel burned by the heavy machines involved in the procedure is also considered. For those subassemblies, the midpoint categories that receive the highest impact are global warming related to human health, fine particulate matter formation, and human carcinogenic toxicity.

The impact of the other midpoint categories, considered separately for each subassembly, does not exceed 5 points. Nevertheless, the ceiling is the subassembly with the highest impact on human non-carcinogenic toxicity (almost 5 points), contributing more than twice compared to the other subassemblies for this category. However, the total pondered impact for this category is about 10 points.

In Figure 5, the contribution, in percentage, of each construction typology (EB and CBB) to the different impact categories from the midpoint assessment point of view is shown.

Notice that results are presented as the percentual contribution of each construction to a common total for each impact category; thus, the sum of both constructions (EB and CBB) adds up to 100%. Figure 5 shows that, in 15 out of 22 impact categories, the impact of the Cement Blocks Building (CBB) is higher than 60%, while this percentage is only surpassed in one case (Land use) for the Earthbag Building.

It is seen that the CBB presents a higher impact than the Earthbag Building (EB) for all the categories but one, the Land use, for which the EB exhibits a higher impact (22.3% higher) compared to the CBB. The higher difference is presented in the human carcinogenic toxicity, 74.5% lower for the EB than the CBB. Other impact categories in which the EB presents a notably lower impact compared to the CBB are those categories related to water ecotoxicity (54.8% lower for freshwater, and 53.6% lower for marine ecotoxicity).

On the other hand, in some other categories, the discrepancies between the two buildings are relatively small (under 15%), such as those related to ozone formation and resource scarcity, where the differences stand at approximately +11.5% and +7.5%, respectively, being the impact higher for the CCB.

Applying the normalization process considered in the ReCiPe 2016 Endpoint methodology, the results are presented in Figure 6. Results are presented in impact points for each construction.

In Figure 7, the final stage of the ReCiPe 2016 Endpoint methodology, the ponderation phase, is presented.

Figure 7 shows the impact produced according to the different categories included in the study, considering the ponderation phase by the two different constructions.

Considering the ponderation results, the impact categories with the highest impacts are fine particulate matter formation, global warming related to human health, and human carcinogenic toxicity.

For the fine particulate matter formation, the contribution of the CBB is 27.9 points whilst for the EB is 17.5 points, a difference of 37%. In the case of global warming related to human health, the results are 29.5 and 14.9 points for the CBB and the EB, respectively, representing a difference of 49.5%.

The categories that present the highest and lowest differences for both stages, with normalization, are presented in Table 4.

As shown in Table 4, after the normalization, the impact produced by the CBB in categories such as human toxicity, both carcinogenic and non-carcinogenic, and ecotoxicity related to water (freshwater and marine), as well as freshwater eutrophication, is more than a 60% higher than the impact produced by the EB. On the other hand, the land use required for the CBB is 57.40% lower than the EB.

Regarding the ponderation results, although for both constructions the highest impact categories are the same, it is noticed that for the CBB the highest impact is presented on the global warming related to human health, and for the EB the highest result is obtained for the fine particulate matter formation.

In Figure 8, the endpoint categories included in the ReCiPe 2016 Endpoint methodology are presented for both buildings.

The endpoint category that receives the highest impact is human health since the midpoint impact categories that contribute to this endpoint category match with those categories that have received the highest impacts, for example, global warming related to human health and fine particulate matter formation.

However, there is a huge difference between both constructions in terms of human health. The CBB has more than twice the impact of the EB, with a result of 92.3 and 40 points, respectively.

As presented in Figure 8, the impact of both constructions regarding the ecosystems or the resources might be dismissed because its impact pondered score is less than 5 points.

Combining the scores across all categories into a total score, the CBB achieves an overall score of 96.1 points, while the EB obtains a total of 42.8 points. This cumulative evaluation reiterates the trend observed in individual categories, with the Cement Blocks Building demonstrating a substantially higher overall score compared to the Earthbag Building.

## 4. Conclusions

Taking into account the results obtained for the two constructions analyzed, Earthbag Building and Cement Blocks Building, with equivalent dimensions but different construction techniques and materials usage, the Cement Blocks Building demonstrates an overall score of 96.1 points, whereas the EB achieves a total of 42.8 points.

The overall environmental impact produced by the Cement Blocks Building is 52.3 points higher than that of the Earthbag Building, representing an increase of 122.3% of the Cement Blocks Building impact compared to that of the Earthbag Building.

Regarding the origin of the materials used in each construction, the environmental impact has a direct relation to the processed materials. The LCA indicates that using highly processed material has a higher impact on the different midpoint and endpoint categories. Thus, the process of the Earthbag Building construction does not require many processed materials and the construction process does not use a huge amount of energy (earth excavation, water pumping, and mortar mixer), so the overall impact is lower than that for the Cement Blocks Building. For the Cement Blocks Building, cement blocks are processed but also the ceiling and the foundation require energy from heavy machinery and cement mortar with reinforced steel for its production, increasing the impact on the environment. For instance, in the case of the Earthbag Building construction, the weight contribution of the steel wire subassembly is 5.11 times less than that of the domo earth mix. Despite this significant difference in individual weights, the overall impact does not scale proportionately. Instead, it is only 2.4 times smaller, indicating that processed materials have a higher impact on the overall construction.

The Earthbag Building emerges as a highly viable choice for dwelling construction, particularly in regions where there is convenient access to abundant raw earth as a construction material, offering a sustainable and resource-efficient solution.

The Earthbag Building not only capitalizes on the availability of earth as a building resource but also presents an eco-friendly alternative reducing the environmental impact in the construction field.

Regarding earth construction, deeper knowledge is required in fields such as thermal comfort, maintenance, and durability. Filling these knowledge gaps will facilitate the promotion of earth construction. More specifically, future work regarding LCA analysis should focus on establishing key performance indicators or functional units to allow comparisons between different construction systems.

## Figures and Tables

**Table 1:** Principal characteristics of the buildings considered in the analysis.

	Earthbag Building	Cement Blocks Building
Type of construction	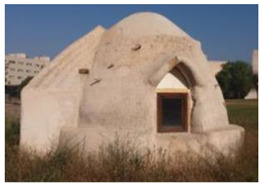	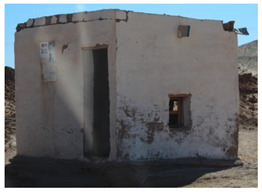
Volume shape	Dome shape	Cube shape
Inner net surface	7.07 m^2^	7.05 m^2^
Height	3.3 m (maximum)	2.5 m
Inner net volume	17.67 m^3^	17.63 m^3^
Door	Exterior frame measuring 0.91 m × 2 m, with a glazed area of 1.09 m^2^ and a wooden frame of 6 cm.	
Windows	Two windows measuring 0.8 × 0.67 m (east) and 0.6 × 0.35 m (west), which represent a glazed area of 0.25 m^2^ and 0.21 m^2^, respectively.	
Main construction materials	Earth mixture consists of 0.80% fine gravel, 92.21% sand, 3.42% slime, and 3.57% clay by weight.	Cement blocks 40 × 20 × 15 cm (BL1550), cement mortar, reinforced steel bars, and aluminum sandwich panels.

**Table 2:** Life Cycle Inventory of the Earthbag Building.

Assembly	Quantity	Unit	Simapro Reference
Clay			
	1498.68	kg	Clay {RoW}|market for clay|APOS, S
Earth/Sand			
	38,709	kg	Shale {GLO}|market for|APOS, S
Lime			
	1435.72	kg	Lime, packed {CH}|market for lime, packed|APOS, S
Domo earth mix			
	1	p	Clay
	1	p	Earth/Sand
	1	p	Lime
	16,792	kg	Water, harvested from rainwater {GLO}|rainwater harvesting|APOS, S
	51	kWh	Electricity, low voltage {ES}|electricity voltage transformation from medium to low voltage|APOS, S
Foundation			
	1000	kg	Gravel, crushed {RoW}|market for gravel, crushed|APOS, S
	752.8	MJ	Diesel, burned in building machine {GLO}|market for|APOS, S
Mastic asphalt			
	20	kg	Mastic asphalt {GLO}|market for|APOS, S
Waterproof PE layer			
	15.24	kg	Polyethylene, high density, granulate {GLO}|market for|APOS, S
PP bags			
	46.2	kg	Polypropylene, high density, granulate {GLO}|market for|APOS, S
Steel wire			
	66	kg	Steel wire rod/EU
Window/Door			
	2.566	m^2^	Glazing, double, U < 1.1 W/m^2^K {RER}|production|APOS, S
	0.022	m^3^	Oriented strand board {RER}|production|APOS, S
	4	kg	Polystyrene foam slab for perimeter insulation {RoW}|processing|APOS, S
	0.0666	m^3^	Glued laminated timber, average glue mix {Europe without Switzerland}|glued laminated timber production, average glue mix|APOS, S
Coating			
	339.3	kg	Water, harvested from rainwater {GLO}|market for water, harvested from rainwater|APOS, S
	1000	kg	Sand {RoW}|market for sand|APOS, S
	300	kg	Lime {RER}|market for lime|APOS, S
	2	kWh	Electricity, low voltage {ES}|electricity voltage transformation from medium to low voltage|APOS, S
Earthbag construction			
	1	p	Domo earth mix
	1	p	Foundation
	1	p	Mastic asphalt
	1	p	Waterproof PE layer
	1	p	PP bags
	1	p	Steel wire
	1	p	Window/Door
	1	p	Coating

**Table 3:** Life Cycle Inventory of the Cement Blocks Building.

Assembly	Quantity	Unit	Simapro Reference
Ceiling			
	67.76	kg	Polystyrene foam slab, 10% recycled {GLO}|market for|APOS, S
	22.032	kg	Aluminium alloy, metal matrix composite {GLO}|market for|APOS, S
Cement blocks walls			
	2596	kg	Concrete block {RoW}|market for concrete block|APOS, S
	622	kg	Cement mortar {RoW}|market for cement mortar|APOS, S
	300	kg	Water, harvested from rainwater {GLO}|market for water, harvested from rainwater|APOS, S
	65.62	kg	Reinforcing steel {GLO}|market for|APOS, S
Foundation			
	1000	kg	Gravel, crushed {RoW}|market for gravel, crushed|APOS, S
	1.5	m^3^	Concrete, 20 MPa {RoW}|market for concrete, 20 MPa|APOS, S
	112.5	kg	Reinforcing steel {GLO}|market for|APOS, S
	752	MJ	Diesel, burned in building machine {GLO}|market for|APOS, S
Window/Door			
	2.566	m^2^	Glazing, double, U < 1.1 W/m^2^K {GLO}|market for|APOS, S
	0.022	m^3^	Oriented strand board {RoW}|market for oriented strand board|APOS, S
	4	kg	Polystyrene foam slab for perimeter insulation {GLO}|market for|APOS, S
	0.0666	m^3^	Glued laminated timber, average glue mix {RoW}|market for glued laminated timber, average glue mix|APOS, S
Cement Blocks Building			
	1	p	Foundation
	1	p	Cement blocks walls
	1	p	Ceiling
	1	p	Window and door

**Table 4:** Midpoint impact categories differences according to the different constructions.

Impact Category Number	Midpoint Impact Category	Normalisation		Ponderation		Impact Difference $\frac{\left(CBB-EB\right)}{CBB}$
		CBB	EB	CBB	EB	
1	Global warming, Human health	7.38 × 10^−2^	3.73 × 10^−2^	29.54	14.93	49.45%
2	Fine particulate matter formation	6.97 × 10^−2^	4.37 × 10^−2^	27.87	17.47	37.32%
3	Human carcinogenic toxicity	6.14 × 10^−2^	8.97 × 10^−3^	24.57	3.59	85.40%
4	Human non-carcinogenic toxicity	2.45 × 10^−2^	9.15 × 10^−3^	9.79	3.66	62.59%
5	Global warming, Terrestrial ecosystems	3.61 × 10^−3^	1.83 × 10^−3^	1.44	7.3 × 10^−1^	49.45%
6	Fossil resource scarcity	5.35 × 10^−3^	4.42 × 10^−3^	1.07	8.8 × 10^−1^	17.45%
7	Water consumption, Human health	1.20 × 10^−3^	6.34 × 10^−4^	4.8 × 10^−1^	2.5 × 10^−1^	47.09%
8	Land use	1.01 × 10^−3^	1.59 × 10^−3^	4.0 × 10^−1^	6.3 × 10^−1^	−57.40%
9	Terrestrial acidification	8.27 × 10^−4^	5.56 × 10^−4^	3.3 × 10^−1^	2.2 × 10^−1^	32.75%
10	Ozone formation, Terrestrial ecosystems	5.36 × 10^−4^	4.23 × 10^−4^	2.1 × 10^−1^	1.7 × 10^−1^	21.04%
11	Freshwater eutrophication	2.91 × 10^−4^	1.14 × 10^−4^	1.2 × 10^−1^	5.0 × 10^−2^	60.90%
12	Ozone formation, Human health	2.26 × 10^−4^	1.80 × 10^−4^	9.04 × 10^−2^	7.21 × 10^−2^	20.24%
13	Water consumption, Terrestrial ecosystem	1.28 × 10^−4^	6.56 × 10^−5^	5.11 × 10^−2^	2.62 × 10^−2^	48.64%
14	Mineral resource scarcity	2.12 × 10^−4^	1.92 × 10^−4^	4.24 × 10^−2^	3.85 × 10^−2^	9.24%
15	Terrestrial ecotoxicity	7.49 × 10^−1^	4.70 × 10^−5^	3.00 × 10^−2^	1.88 × 10^−2^	37.30%
16	Freshwater ecotoxicity	5.52 × 10^−5^	1.61 × 10^−5^	2.21 × 10^−2^	6.44 × 10^−3^	70.85%
17	Ionizing radiation	1.93 × 10^−5^	1.14 × 10^−5^	7.73 × 10^−3^	4.57 × 10^−3^	40.85%
18	Marine ecotoxicity	1.14 × 10^−5^	3.45 × 10^−6^	4.57 × 10^−3^	1.38 × 10^−3^	69.78%
19	Stratospheric ozone depletion	1.07 × 10^−1^	8.65 × 10^−6^	4.27 × 10^−3^	3.46 × 10^−3^	18.99%
20	Global warming, Freshwater ecosystems	9.87 × 10^−4^	4.99 × 10^−8^	3.95 × 10^−6^	1.99 × 10^−5^	49.45%
21	Marine eutrophication	5.48 × 10^−8^	2.65 × 10^−8^	2.19 × 10^−6^	1.06 × 10^−5^	51.73%
22	Water consumption, Aquatic ecosystems	1.06 × 10^−8^	4.54 × 10^−9^	4.25 × 10^−6^	1.82 × 10^−6^	57.30%

## Data Availability

Data are contained within the article.
